# Analysis of Punicalin and Punicalagin Interaction with PDIA3 and PDIA1

**DOI:** 10.3390/ijms251910531

**Published:** 2024-09-30

**Authors:** Giorgia Meschiari, Marco Minacori, Sara Fiorini, Mariassunta Tedesco, Margherita Eufemi, Fabio Altieri

**Affiliations:** 1 Department of Biochemical Science “A. Rossi Fanelli”, Faculty of Farmacy and Medicine, Sapienza University of Rome, Pl. A. Moro 5, 00185 Rome, Italy; giorgia.meschiari@uniroma1.it (G.M.); sara.fiorini@uniroma1.it (S.F.); tedescomariassunta@gmail.com (M.T.); margherita.eufemi@uniroma1.it (M.E.); 2 Department of Bioscience and Agro-Food and Environmental Technology, University of Teramo, Campus “Aurelio Saliceti”, Via R. Balzarini 1, 64100 Teramo, Italy; marco.minacori@uniroma1.it

**Keywords:** protein disulfide isomerase, punicalin, punicalagin, PDIA3, PDIA1, ERp57, inhibitor, fluorescence quenching analysis, glioblastoma

## Abstract

PDIA3 is a pleiotropic protein primarily located in the endoplasmic reticulum where it is involved in protein folding, catalyzing the formation, breakage, and rearrangement of disulfide bonds. PDIA3 is implicated in numerous pathologies such as cancer, inflammation, and neurodegeneration. Although punicalagin has been proven to be a highly promising PDIA3 inhibitor and can be used as target protein in glioblastoma, it does not have sufficient selectivity for PDIA3 and is a quite-large molecule. With the aim of finding punicalagin derivatives with a simplified structure, we selected punicalin, which lacks the hexahydroxy-diphenic acid moiety. Previous docking studies suggest that this part of the molecule is not involved in the binding with PDIA3. In this study we compared the ability of punicalin to bind and inhibit PDIA3 and PDIA1. Tryptophan fluorescence quenching and disulfide reductase activity (using both glutathione and insulin as substrates) were evaluated, demonstrating the ability of punicalin to bind and inhibit PDIA3 even to a lesser extent compared to punicalagin. On the other hand, punicalin showed a very low inhibition activity towards PDIA1, demonstrating a higher selectivity for PDIA3. Protein thermal shift assay evidenced that both proteins can be destabilized by punicalin as well as punicalagin, with PDIA3 much more sensitive. Additionally, punicalin showed a higher change in the thermal stability of PDIA3, with a shift up to 8 °C. This result could explain the presence of PDIA3 aggregates, evidenced by immunofluorescence analysis, that accumulate within treated cells and that are more evident in the presence of punicalin. The results here obtained show punicalin is able to bind both proteins but with a higher selectivity for PDIA3, suggesting the possibility of developing new molecules with a simplified structure that are still able to selectively bind and inhibit PDIA3.

## 1. Introduction

Protein disulfide isomerases (PDIs) are an important cellular oxidoreductase enzyme family includes more than 20 members characterized by thioredoxin-like (TRX-like) domains, which show differences in structure, size, tissue distribution, and enzymatic activity [1,2]. PDIs are primarily located in the endoplasmic reticulum (ER), where they are implicated in protein folding, catalyzing the formation, breakage, and rearrangement of disulfide bonds by the presence of one or more redox-active sites involving the canonical Cys–X–X–Cys sequence [3,4,5]. PDIs may also act as molecular chaperones as they are capable of distinguishing between native, unfolded, or misfolded proteins through hydrophobic interactions [6,7,8]. PDIA1 and PDIA3 are the main characterized members, sharing a high degree of structural homology. Both have a molecular weight of about 57 KDa and are characterized by four TRX-like domains organized in a U-shaped scaffold [9]. The first and the fourth domains (a and a’ domains) hold a TRX-like active site containing the CGHC sequence, which provides redox activity, while the central catalytically inactive domains (b and b’ domains) provide binding and folding of target proteins [10]. The two isoforms are mainly located in the ER, participating in the correct folding of protein with the primary PDIA3 involved in the quality control of neo-synthesized glycoproteins [11,12]. PDIA1 is involved in ER stress and the unfolded protein response (UPR) pathway [13,14], while PDIA3 is a pleiotropic protein found also in the cytosol, in the nucleus, and on the cell surface, and is involved in multiple processes [15].

Although some PDIs (i.e., PDIA1) are expressed at high levels in normal cells, to maintain the proper protein folding and/or in response to UPR signaling, several studies have highlighted that PDIA1 and PDIA3 expression is upregulated in various cancers and neurodegenerative diseases [16,17,18,19,20].

Several studies highlight that PDIA3 expression is upregulated in various cancers and tightly associated with the occurrence, development, invasion, and metastasis of tumor cells. Cancer is a major burden of disease worldwide, and in 2022 there were an estimated 20 million new cancer cases [21]. PDIA3 is overexpressed in most cervical cancers [22], malignant stages of prostate cancer [23], uveal melanoma [24], gliomas [25], clear cell renal cell carcinoma [26], and hepatocellular carcinoma (HCC) [27]. Zhang and collaborators have demonstrated that PDIA3 is involved in inflammation, interaction with other immune checkpoint inhibitors, and suppression of anti-tumor immunity in the glioma microenvironment [28].

The therapeutic potential of PDI inhibitors in cancer treatment is promising, and numerous PDI inhibitors have been identified through chemical screening of synthetic compounds and natural products [29,30]. However, none of the identified molecules is currently approved by FDA for cancer treatment. One of the major problems is that most of the inhibitors developed were obtained by screening studies primarily focused on PDIA1. The presence of several PDI isoforms, with overlapping substrate specificities, may represent a critical point in the development of PDI inhibitors as a therapeutic target in cancer.

**Figure 1:**
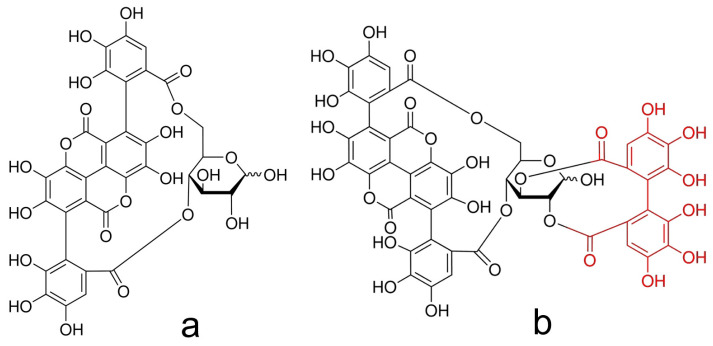
Molecular structure of punicalin (**a**) and punicalagin (**b**). The hexahydroxydiphenic acid moiety is shown in red.

The specific biomolecular interactions [31] between PDIA3 and certain flavonoids can affect the protein reductase activity, and thus act as a potential therapeutical agent against cancer diseases [32]. We have identified punicalagin as a PDIA3 inhibitor [33], and in a recent work [34] we provide evidence that PDIA3 inhibition offers a valid therapeutic treatment in glioblastoma, confirming the importance of identifying selective inhibitors. Punicalagin is an ellagitannin mostly present in pomegranate peel (Punica granatum) [35], and its anticancer, anti-atherosclerotic, anti-obesity, neuroprotective, and anti-inflammatory properties are already known [36,37,38,39]. Although punicalagin has been proven to be a highly promising PDIA3 inhibitor, it does not have a sufficient selectivity for PDIA3 and is a quite-large molecule. For this reason, we decided to compare punicalagin with punicalin, a molecule having a simplified structure compared to punicalagin (Figure 1).

Punicalin lacks the hexahydroxydiphenic acid moiety which, based on previous docking data, should not be involved in the binding with PDIA3 but only with PDIA1 [40]. Thus, the ability of punicalin, as well as punicalagin, to bind and inhibit PDIA3, has been evaluated and compared to PDIA1, the most abundant member of the PDI family, and which shares with PDIA3 a considerable similarity in structure and enzymatic functions.

## 2. Results

### 2.1. Analysis of Punicalin–PDI Interaction by Fluorescence Quenching

**Figure 2:**
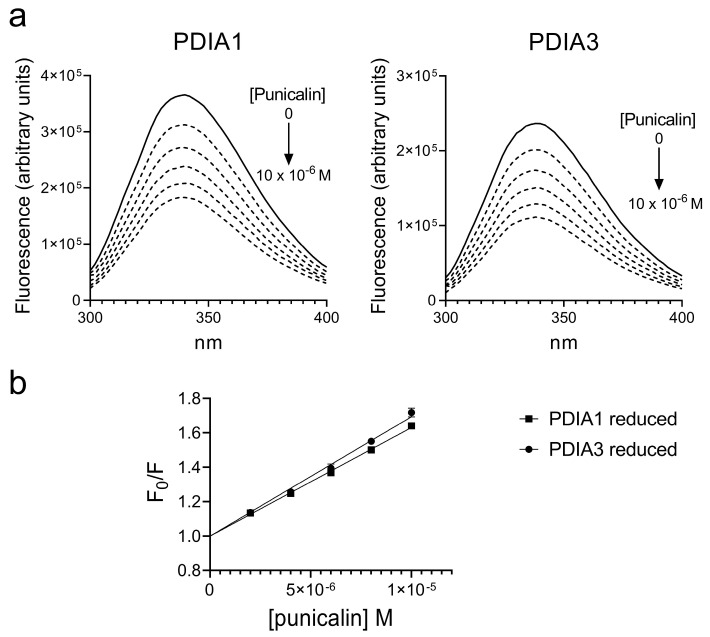
(**a**) Fluorescence quenching spectra of reduced PDIA1 and PDIA3 alone (solid line) and after stepwise addition of punicalin (dotted line) (pH 7.4, 25 °C, and λ_ex_ = 290 nm). (PDI) = 0.1 × 10^−6^ M, (punicalin) = 10 × 10^−6^ M final concentration); (**b**) Stern–Volmer plot of quenching data of reduced PDIs in the presence of increasing concentrations of punicalin.

In a previous article, we evaluated the ability of punicalagin to interact with PDIA1 and PDIA3 [40]. In this study, the results previously obtained were compared with those obtained using punicalin. The protein–ligand interaction was evaluated by intrinsic quenching analysis due to the presence, in both proteins, of aromatic amino acids, among which tryptophan residues represent the main source. Quenching analysis was performed on PDIA1 and PDIA3, in the reduced form, by adding increasing concentrations of punicalin and recording the fluorescence spectra of both proteins. The fluorescence intensity of both proteins decreased in the presence of punicalin, suggesting a protein–ligand interaction near the tryptophan residues (Figure 2). Quenching of intrinsic tryptophan fluorescence of both proteins was assessed using the Stern–Volmer equation, as previously reported [40]. To further characterize the binding process, fluorescence data were analyzed using the equation described by Bi et al. [41] and the reiterative calculation process described by Sun et al. [42], providing an estimated value of the dissociation constant (K_d_). The values of the Stern–Volmer constant (K_SV_) and the K_d_ obtained for punicalin are summarized in Table 1 and compared with those obtained for punicalagin [40]. The comparison of the results confirms that punicalin can bind both PDIs, although showing a slightly lesser affinity with respect to punicalagin.

### 2.2. Punicalin Effect on PDIA Reductase Activity

The enzymatic activity of PDIA proteins was evaluated by glutathione reduction and insulin turbidity assays. PDIA1 and PDIA3 are proteins capable of catalyzing oxidation-reduction and isomerization reactions through thiol groups and disulfide bonds. Because the action of punicalagin on the reductase activity of the two proteins was previously evaluated by glutathione reduction assay [40], the same assay was repeated using punicalin to compare the effect of both molecules on PDIs.

**Figure 3:**
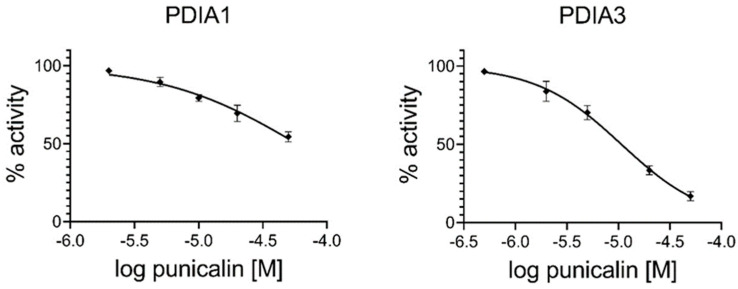
Dose–response curve of punicalin showing the inhibition of PDIA1 and PDIA3 reductase activity using di-E-GSSG as substrate. Data were calculated using both proteins (20 nM) under reducing conditions and increasing concentrations (from 2 µM to 50 µM) of punicalin.

The graphs in Figure 3 report the percentage of residual activity of each PDI as a function of the logarithm of punicalin concentration, and based on these data the IC_50_ values were evaluated and compared with those previously obtained for punicalagin [40] (Table 2). Punicalin, as reported for punicalagin, preferably inhibits PDIA3 compared to PDIA1, although with a lower inhibitory extent. In fact, the IC_50_ values estimated for punicalin and punicalagin toward PDIA3 were 11.0 × 10^−6^ M and 1.5 × 10^−6^ M, respectively. The IC_50_ values estimated for punicalin and punicalagin toward PDIA1 were instead 58.0 × 10^−6^ M and 6.1 × 10^−6^ M, respectively (Table 2).

**Figure 4:**
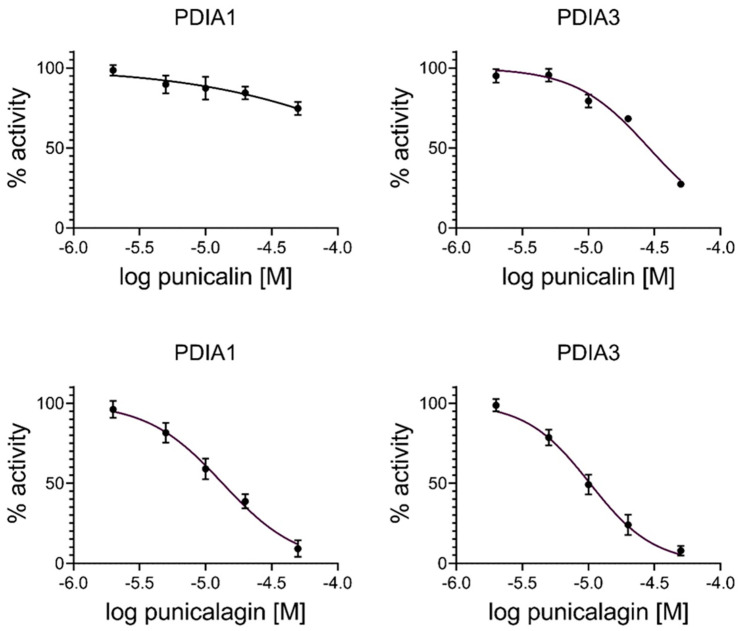
Dose–response curve of punicalin (**upper panel**) and punicalagin (**lower panel**) showing the inhibition of PDIA1 and PDIA3 reductase activity using insulin as a substrate. Data were calculated using both proteins under reducing conditions (PDIA1 800 nM and PDIA3 1600 nM) and increasing concentrations (from 2 µM to 50 µM) of punicalagin and punicalin.

To further evaluate the inhibitory effect of punicalin and punicalagin on the enzymatic activity of the two proteins, we performed a different assay using insulin, a physiologically more appropriate substrate. The results obtained are reported in Figure 4 and show again a lesser inhibitory effect of punicalin compared to punicalagin, as observed with the glutathione-based assay. However, the inhibition activity of punicalin on PDIA1 is very low, with an IC_50_ value > 250.0 × 10^−6^ M (Table 3).

### 2.3. Effect of Punicalin on T98G Cells

**Figure 5:**
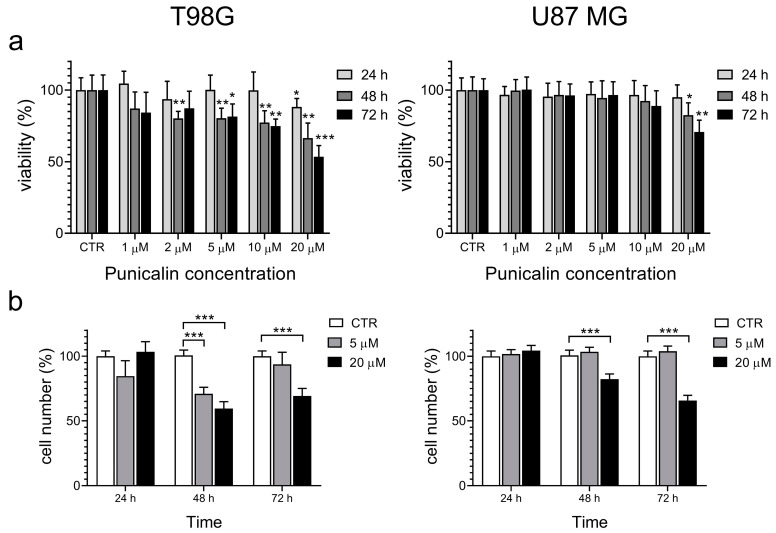
Effect of punicalin treatment on T98G (left) and U87 MG (right) cells. (**a**) Cell viability by MTT assay. Cells were treated with punicalin (from 1 µM to 20 µM) for 24 h, 48 h, and 72 h. Control untreated cells were analyzed at the same time points. Experiments were repeated three times, and the obtained results were reported as the mean and SEM. (**b**) Cell number. Cells were treated with punicalin (5 µM and 20 µM) for 24 h, 48 h, and 72 h. Control untreated cells were analyzed at the same time points. For each time point, the % of cell number refers to the control untreated cells at the same time. Experiments were repeated three times, and the obtained results are reported as the mean and SEM. Statistical analysis was performed using a two-tailed Student’s *t*-test comparing each point with the corresponding untreated control (* *p* < 0.05, ** *p* < 0.01, *** *p* < 0.001).

The cell viability assay was used to evaluate the effect of punicalin as a PDI inhibitor. T98G and U87 MG glioblastoma cell lines were treated with increasing concentrations of punicalin, and the reduction in MTT substrate was determined after 24 h, 48 h, and 72 h. Previously we demonstrated that punicalagin shows a cytotoxic effect in glioblastoma cell lines, especially in T98G cells [34]. Here, we compared the ability of punicalin with that of punicalagin to prevent the growth of these cells (Figure 5).

Punicalin, like punicalagin, caused a concentration- and time-dependent decrease in cell viability on the T98G cell line (Figure 5a, left panel). In general, the effect of punicalin is slightly lower compared to that observed for punicalagin at the same concentration [34]. The cell viability of U87 MG cells is less affected by punicalin treatment, and the effect becomes evident only at the highest concentration (Figure 5a, right panel). This may be the result of differences in the two cell lines. We previously reported a lower effect of punicalagin treatment on the viability of U87 MG cells compared to the T98G cells. Moreover, the lower expression level of PDIA3 reported in the U87 MG cell line [34] and the higher selectivity of punicalin for PDIA3 compared to PDIA1 may contribute to the reduced effect of punicalin on the viability of U87 MG cells.

The number of cells was evaluated after 24 h, 48 h, and 72 h of treatment with punicalin at the concentration of 5 µM and 20 µM (Figure 5b). A marked reduction in the T98G cell number was observed after 48 h of treatment at both concentrations (Figure 5b, left panel). However, an increase in the cell number occurred after 72 h of treatment with punicalin at a 5 µM concentration. This result was not evident when cells were treated with punicalin at a 20 µM concentration. Based on these results, punicalin has a preferential cytostatic effect at a low concentration (5 µM), becoming cytotoxic at a 20 µM concentration. Punicalin treatment also negatively affects the U87 MG cell number but, similar to what was observed for cell viability, the effect is evident only at the highest concentration used (Figure 5b, right panel).

### 2.4. Analysis of the Effect of Punicalin and Punicalagin on Protein Stability by Differential Scanning Fluorimetry

The effect of punicalin and punicalagin on the stability of both proteins was evaluated by differential scanning fluorimetry (DSF). Initially, PDIA1 and PDIA3 were incubated at 25 °C for 20 min with both substances at 5 µM and 20 µM concentrations. The initial temperature of the instrument plate was set at 25 °C and gradually increased up to a maximum of 70 °C. The reading was optimized at intervals of 0.2 °C every 5 s. Once the fluorescence profile was obtained, the data were analyzed with the GraphPad prism program, by either fitting data with a Boltzmann equation or calculating the first derivative. In DSF analyses, a gradual increase in fluorescence is observed, due to a conformational change in the protein, which reaches a maximum value and then decreases because of protein precipitation.

**Figure 6:**
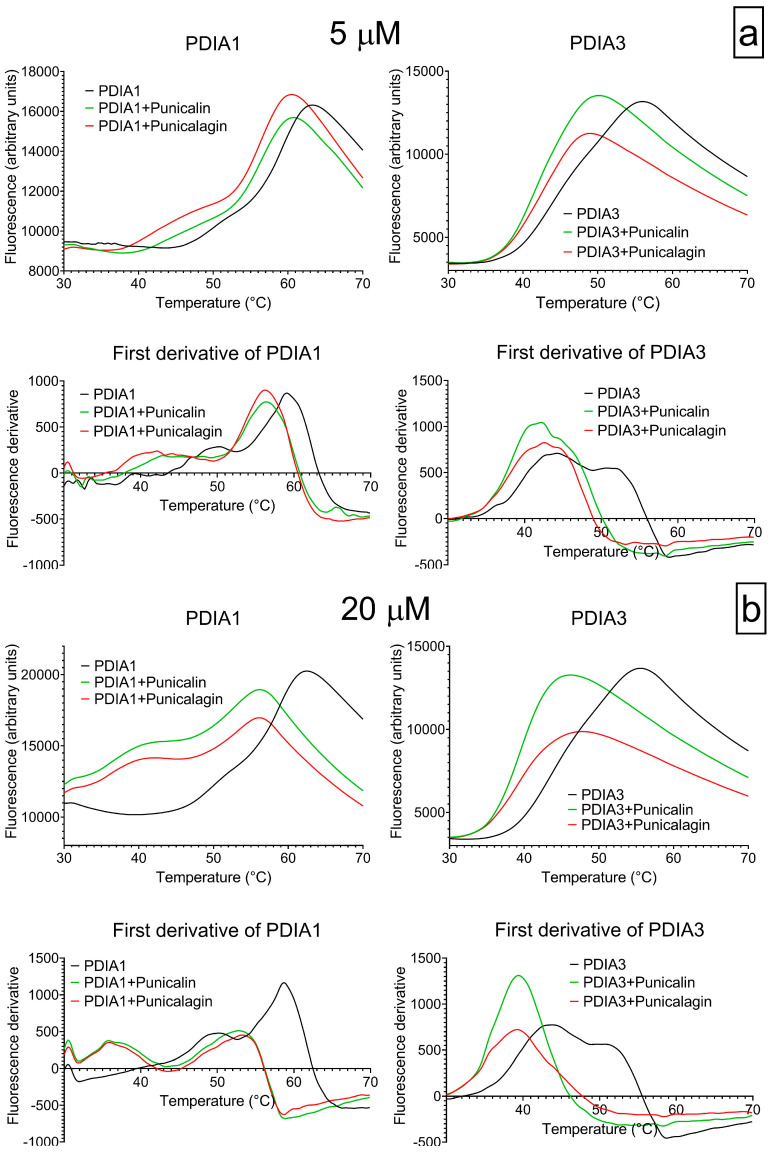
The thermal stability of PDIA1 and PDIA3 was evaluated by DSF in the presence of punicalagin and punicalin at concentrations of 5 µM (panel **a**) and 20 µM (panel **b**). In each panel, the graphs in the first row show the syprorange fluorescence change as a function of the temperature for PDIA3 and PDIA1 alone or in the presence of the two substances; the graphs in the second row report the first derivative of the same data.

The results show the ability of both substances to affect protein stability with a marked effect on PDIA3 (Figure 6). The first-derivative graph shows the presence of two overlapped transitions in the range 35–55 °C for PDIA3 and a pre-transition followed by a large one in the range 45–65 °C for PDIA1. The two transitions may represent different denaturation steps in which, respectively, partial or complete protein denaturation occurs, as previously observed by differential scanning calorimetry [40]. The first transition may represent an early opening of the protein with the switching from a closed to an opened conformation and a different distance between the a and a’ domains.

The addition of punicalin or punicalagin resulted in a change in the fluorescence thermal profile with a shift towards a lower temperature of all transitions. The effect became more evident in the presence of punicalin/punicalagin at a 20 µM concentration. Considering the first-derivative graph, in the case of PDIA1, the shift is larger for the pre-transition, while is about 5 °C for the second main transition. The effect on PDIA3 is quite significant, with a shift of more than 10 °C for the second transition; this is much closer to the first one, which is left shifted by about 3 °C (Figure 6).

**Figure 7:**
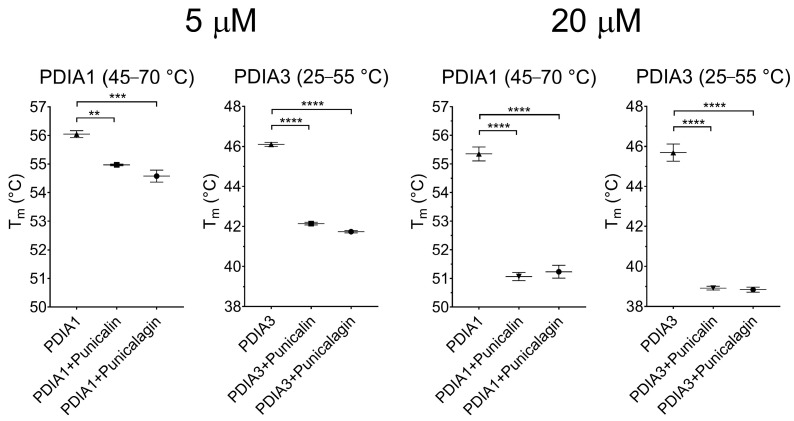
T_m_ values evaluated from the fluorescence data by fitting selected range of temperature with a Boltzmann equation. Plots are displayed as mean and standard deviations of at least three independent measurements. Statistical analysis was performed using a two-tailed Student’s *t*-test comparing each point with the corresponding untreated control (** *p* < 0.01, *** *p* < 0.001, **** *p* <0.0001).

To evaluate T_m_, we focused the attention on the main transition, and thus we fitted, with a Boltzmann equation, the data in the range of 25–55 °C for PDIA3 and in the range of 45–70 °C for PDIA1. Again, the two substances show a destabilizing effect, which was more evident for PDIA3 than for PDIA1. In fact, punicalagin and punicalin at a 20 µM concentration cause a shift of T_m_ of at least 6 °C in the case of PDIA3, while this is approximately 3 °C for PDIA1 (Figure 7).

It is important to note the different thermal stability of the two proteins evidenced by this technique, which is much lower for PDIA3. In fact, the T_m_ value calculated for PDIA1 is around 56 °C, while that for PDIA3 is 46 °C. Moreover, the addition of punicalin/punicalagin lowers the T_m_ of PDIA3 below 40 °C, making some destabilizing effects possible, even at physiological temperature.

The data obtained so far indicate the ability of punicalagin and punicalin to bind, inhibit, and induce a conformational change, mainly on PDIA3, which causes exposure hydrophobic patches, which may promote protein aggregation.

### 2.5. Analysis of Cellular Distribution of PDIA3 by Immunofluorescence

All comparative data obtained so far suggest the preferential inhibition of punicalin and punicalagin towards PDIA3 compared to PDIA1, with punicalin much more selective.

**Figure 8:**
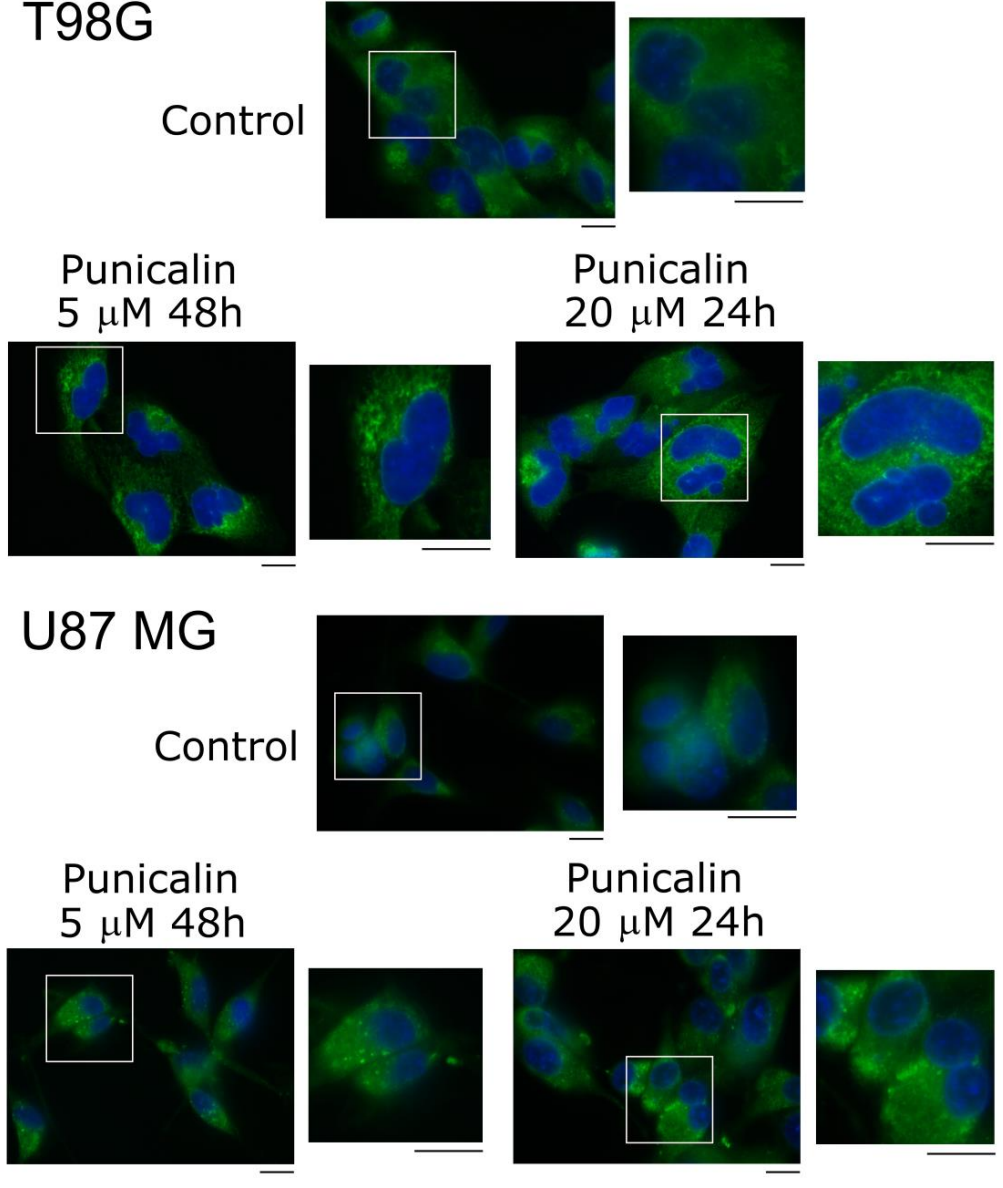
Cellular distribution of PDIA3 upon punicalin stimulation visualized through immunofluorescence staining of PDIA3: T98G and U87 MG cells were subjected to punicalin treatments of 5 µM for 48 h and 20 µM for 24 h. Control corresponded to untreated cells. PDIA3 was stained in green and the nuclei counterstained in blue using DAPI. Images reported are representative of three independent experiments and were captured under the same acquisition parameters. Insets show enlarged portions of the acquired images. Images were captured with a confocal fluorescence microscope (Leica AF 6000) using a 60× oil immersion objective (scale bar 25 μm).

Possible changes in the cellular distribution of PDIA3 were previously studied by immunofluorescence assay. Recently, Bilches Medinas et al. [43] reported that a PDIA3 mutation with the loss of functions induced protein aggregation. We reported that punicalagin, an inhibitor of the enzymatic activity of PDIA3, induced the formation of protein aggregates 24 h after treatment with a concentration of 30 µM [34]. Based on these results, we decided to verify whether punicalin, like punicalagin, was able to determine changes in the cellular distribution of PDIA3. T98G and U87 MG cells were treated with 5 µM and 20 µM of punicalin for 48 h and 24 h, respectively (Figure 8).

Punicalin treatment, as previously reported for punicalagin [40], caused a marked change in the fluorescence pattern and a cellular redistribution of PDIA3, suggesting the formation of protein aggregates. The effect is visible in both cell lines, and in U87 MG cells seems more evident than that previously observed for punicalagin. This could be related to the greater selectivity of punicalin towards PDIA3 compared to punicalagin. Furthermore, based on the data obtained with the DSF, we can hypothesize that the formation of the protein aggregates induced by the two substances may be due not only to their ability to bind and inhibit PDIA3, but also to the fact that, even at physiological temperature, they can cause an exposure of hydrophobic residues with the consequent aggregation of the protein.

## 3. Discussion

The ability of punicalagin to bind and inhibit PDIA1 and PDIA3 was previously evaluated. Punicalagin binds both proteins with a similar affinity but shows a greater inhibitory capacity towards PDIA3. In this work, we evaluated the affinity and the effect of punicalin, an ellagitannin related to punicalagin but with a reduced size, towards both PDIAs and compared to punicalagin.

Punicalin was able to better quench PDIA3 intrinsic fluorescence compared to PDIA1, indicating a greater affinity, and showed a greater selectivity to inhibit the disulfide reductase activity of PDIA3 compared to PDIA1. Punicalin, like punicalagin, caused a concentration- and time-dependent decrease in cell viability of the T98G cell line. Treatment with 20 µM punicalin significantly reduced the cell viability, generating a strong cytotoxic effect, comparable to what was previously observed for punicalagin. Despite lacking the hexahydroxy-diphenic acid moiety, punicalin yielded a similar effect compared to punicalagin, but with an increased selectivity towards PDIA3. In addition, both inhibitors caused a marked cellular redistribution of PDIA3 following treatment with 20 µM, with the formation of cytosolic aggregates of protein. However, the effect was more evident with punicalin, and this could be related to its greater selectivity towards PDIA3. Furthermore, based on the data obtained with DSF analysis, we can hypothesize that the formation of the protein aggregates induced by the two substances may be due not only to their ability to bind and inhibit PDIA3, but also to the fact that at physiological temperature they cause an exposure of hydrophobic residues with the consequent aggregation and precipitation of the protein.

In a recent work, we tempted to dock punicalagin within the structure of PDIA1 and PDIA3 by a computational approach [40]. Two different binding sites were evidenced on the two proteins, in which punicalagin was docked with a different orientation. Based on these data, the hexahydroxy-diphenic acid moiety of punicalagin played a major role in the binding to PDIA1, while it does not seem to be involved in the interaction with PDIA3. Thus, the results obtained here, which show that punicalin is able to bind both proteins, but with a higher selectivity for PDIA3, are consistent with the docking data and suggest the possibility of developing new molecules with a simplified structure that are still able to selectively bind and inhibit PDIA3.

## 4. Materials and Methods

### 4.1. Chemicals

Punicalagin and punicalin were obtained from Sigma-Aldrich (Milan, Italy). Reagents used in this study, unless otherwise stated, were bought from Sigma-Aldrich (Milan, Italy). EDTA (ethylenediamine tetra-acetic acid) 0.5 M solution pH 8.0 was bought from IBI Scientific (Milan, Italy), and Tris(hydroxymethyl)aminomethane for buffer solutions from Merck Millipore (Milan, Italy).

### 4.2. Recombinant Proteins: Production and Purification

The mature recombinant form (devoid of the N-terminal presence) of the human PDIA3 protein was expressed and purified according to the procedure previously described [32]. The mature human PDIA1 with an N-terminal His6x-tag was expressed and purified according to the procedure described by Paglia et al. [40]. In both cases, protein purification was evaluated by SDS-PAGE and Coomassie Blue staining, while the concentration was spectrophotometrically estimated using the extinction coefficient (PDIA3 in the reduced form, ε_280_ = 44,810 M^−1^cm^−1^; PDIA1 in the reduced form, ε_280_ = 45,567 M^−1^cm^−1^).

### 4.3. Measurement of Protein Fluorescence Quenching

Protein–ligand interactions were evaluated by titration of intrinsic fluorescence quenching. Protein mission spectra were recorded by a SPEX-FluoroMax spectrofluorometer (Horiba Scientific, Piscataway, NJ, USA) from 300 to 400 nm, with an excitation wavelength set at 290 nm, emission slit set width, a 2 nm bandpass, and a scan speed set at 120 nm min^−1^. PDIA1 and PDIA3 stock solutions (50 µM) were reduced by adding DTT 5 mM. Aliquots of freshly reduced proteins were diluted (0.25 µM final concentration) in phosphate-buffered saline (PBS) pH 7.4 containing 0.2 mM EDTA and 0.1 mM DTT in a 10 mm path length quartz fluorescence cuvette under continuous stirring. The titrations were performed by stepwise additions, at 5 min time intervals of punicalagin/punicalin aliquots (1 mM in PBS pH 7.4 freshly prepared from a 5 mM stock solution in water) to reach a final concentration ranging from 0 to 10 µM. Blank spectra for each experiment (no protein added) were performed in parallel for background determination. The emission spectrum of the protein was recorded and the fluorescence intensities at 338 nm, obtained in at least three independent titration experiments, were used for quenching analyses, as previously described [32]. Quenching of intrinsic tryptophan fluorescence at 338 nm was analyzed by the Stern–Volmer equation (F_o_/F = 1 + K_SV_[L]), where F and F_o_ are the fluorescence intensity, respectively with or without ligand (L), and K_SV_ is the Stern–Volmer constant value. Fluorescence data at 338 nm were also analyzed using the equation described by Bi et al. [41] and the reiterative calculation process described by Sun et al. [42], providing an estimated value of K_d_.

### 4.4. Measurement of PDIAs Disulfide Reductase Activity

#### 4.4.1. Glutathione Reduction Assay

Di-eosin glutathione disulfide (di-E-GSSG) was synthesized by the reaction of eosin isothiocyanate with oxidized glutathione (GSSG) and used as a fluorogenic substrate of PDIs to monitor disulfide reductase activity according to the method of Raturi and Mutus [44], with some modifications [33]. Protein activity was evaluated by monitoring the emission fluorescence increase (λ_em_ = 545 nm and λ_ex_ = 525 nm), and the effect of punicalin (concentration ranging from 2 to 50 µM) was tested.

#### 4.4.2. Insulin Turbidity Assay

PDI disulfide reductase activity was assayed by measuring the reduction of insulin catalyzed by PDIs with DTT. A 50 μM stock solution of each PDI was reduced using TCEP (5 mM final concentration) for 90 min at room temperature. Aliquots of the stock solution were diluted in the incubation mixture (PDIA1 1.6 µM and PDIA3 3.2 µM) containing sodium phosphate buffer (100 mM sodium phosphate, 2 mM EDTA, pH 7.0) added with punicalin/punicalagin (concentration ranging from 0 to 50 µM) and incubated at 37 °C for 1 h. After incubation, an equal volume of the 2× reaction mixture (consisting of DTT 50 μM and human insulin 0.32 mM) was added. Protein activity was evaluated by monitoring the increase in turbidity of the solutions at 600 nm.

In both assays, the inhibition constants were extrapolated by GraphPad Prism 8.0 software (GraphPad Software, San Diego, CA, USA), plotting the obtained data as logarithm dose–response curves.

### 4.5. Differential Scanning Fluorimetry

Protein stability in the absence and presence of punicalagin/punicalin was tested by means of the fluorescence-based thermal stability assay or differential scanning fluorimetry (DSF) developed by Pantoliano and coworkers [45]. DSF records the fluorescence emission signal from the binding of a dye (SYPRO orange) to the exposed hydrophobic patches upon protein unfolding. Fluorescence measurements were performed using a CFX96 Connect RT-PCR instrument (BioRad, Hercules, CA, USA). Aliquots of 18 μL of protein solution (2 μM), freshly diluted in Tris Buffered saline (50 mM TrisHCl, pH 8.0, 150 mM NaCl) containing 0.1 mM DTT, 0.2 mM EDTA, and 1: 1000 dilution of SYPRO Orange (50 mM stock solution in DMSO), were distributed in a 96-well plate. Sample wells were added with punicalagin or punicalin at a final concentration of 5 uM or 20 μM by adding 2 μL of a 10-fold concentrated freshly prepared stock solution. As control, the protein solution was added with 2 μL of the dilution buffer. A melting point analysis was recorded by increasing the temperature from 25 to 70 °C with an increment of 0.2 °C every 5 s and reading, at each temperature, the fluorescence in fluorescence resonance energy transfer mode (FRET). Each measure was the average of three sample well readings, and each DSF experiment was repeated at least three times. To calculate T_m_ values, DSF data from the melting curve were exported and fitted to a Boltzmann equation using GraphPad software [46]. The effect of punicalagin and punicalin was evaluated comparing the T_m_ values in the presence and absence of each molecule.

### 4.6. Cell Cultures

Human glioblastoma cell lines T9 and U-87 MG were obtained from the American Type Culture Collection (ATCC, Manassas, VA, USA). Cells were grown to 70% confluence at 37 °C in 5% CO_2_ in DMEM-HG medium (Sigma-Aldrich, Milan, Italy, cat. D5671) supplemented with 1% sodium pyruvate (Sigma-Aldrich, Milan, Italy, cat. S8636), 10% fetal bovine serum (FBS) (Sigma-Aldrich, Milan, Italy, cat. F7524), 2 mM glutamine (Sigma-Aldrich, Milan, Italy, cat. G7513), and 100 μg/mL streptomycin and 100 U/mL penicillin (Sigma-Aldrich, Milan, Italy, cat. P4333). Punicalagin (Sigma-Aldrich, Milan, Italy, cat. P0023, purity > 98%) and punicalin (Sigma-Aldrich, Milan, Italy, cat. P0023, purity > 98%) dissolved in water, were tested on the T98G cell line.

### 4.7. Viability Assay

T98G and U-87 MG cells were seeded into 96-well plates (10.000 cells/well). The effect of punicalin, at the final concentration of 1 µM to 20 µM, was evaluated on cell viability after 24 h and 48 h of incubation. To assess cell viability, the enzymatic reduction of MTT (3-(4,5-dimethylthiazol-2-yl)-2,5-diphenyl-2H-tetrazolium bromide) (Sigma-Aldrich, Milan, Italy, cat. M2128) to MTT formazan was used. Cell cultures were further incubated for 2 h in culture medium containing 0.5 mg/mL MTT. After 2 h of incubation, the solution was removed and the blue MTT formazan product was dissolved in DMSO (Sigma-Aldrich, cat. D8418). After 30 min at room temperature, the absorbance of the formazan solution was spectrophotometrically read at 570 nm using an Appliskan^®^ plate reader (Thermo Fisher Scientific, Monza, Italy).

### 4.8. Cell Counting

T98G and U-87 MG cells were seeded into 12-well plates (70,000 cells/well) and treated with 5 µM and 20 µM concentrations of punicalin for 24 h, 48 h, and 72 h. At the end of incubation, the cells were washed with PBS (Sigma-Aldrich, cat. D8662), fixed with cold methanol for 20 min, stained with 0.1% crystal violet in methanol/PBS (ratio 1:4) at room temperature for 1 h, and washed again in PBS. To estimate the number of cells remaining in each well, the crystal violet was solubilized by adding a methanol/water solution (50% *v*/*v*) and the absorbance at 595 nm was measured.

### 4.9. Immunofluorescence

T98G and U-87 MG cells were seeded on coverslips into 6-well plates (200,000 cells/well) and incubated for 24 h and 48 h in the presence of 5 µM and 20 µM punicalagin/punicalin. Cells grown on coverslips were washed with PBS, fixed with 3.7% formaldehyde for 20 min, rinsed with PBS, and then permeabilized with cold methanol (−20 °C) for 5 min. Immunofluorescence analysis was performed according to Paglia et al. [40] using a specific primary antibody against PDIA3 (Millipore, Milan, Italy, cat. ABE1032). Coverslips were mounted on glass microscope slides with DuolinkTM mounting medium and examined using a fluorescence microscope (Leica AF6000 Modular System, Leica, Milan, Italy) with a 63× oil immersion objective. Samples were captured under the same acquisition parameters.

### 4.10. Statistics

All statistical analyses were performed with GraphPad Prism 8.0 software (GraphPad Software, San Diego, CA, USA). Comparisons between groups for statistical significance were assessed using multiple t-test analysis. A *p*-value < 0.05 was considered statistically significant and all data presented were the result of at least three independent experiments.

## 5. Conclusions

Although the therapeutic potential of PDI inhibitors in cancer treatment is promising, most of the molecules identified lack selectivity toward the different PDI isoforms. We previously evidenced punicalagin as a promising PDIA3 inhibitor, and we compared its interaction with PDIA1 and PDIA3. In this work, we analyzed punicalin, a derivative of punicalagin with a smaller size and lacking the hexahydroxy-diphenic acid moiety that, from computational studies, does not seem to be involved in the interaction with PDIA1. Consistent with this hypothesis, punicalin proven to be a more selective inhibitor of PDIA3, suggesting the possibility of developing new molecules based on the ellagitannin structure that are able to selectively bind and inhibit PDIA3. Additionally, DSF studies indicate that punicalin and, to a lesser extent, punicalagin, negatively affect the thermal stability of PDIA3, probably inducing a conformational change that causes the exposure of hydrophobic patches and may promote protein aggregation.

The results of this work, even if mostly based on in vitro analyses, confirm punicalin as a suitable PDIA3 inhibitor and lay the basis for further studies, based on the punicalin scaffold, to find similar and even smaller structures with improved affinity and selectivity for PDIA3 and enhanced pharmacodynamic and bioavailability.

## Figures and Tables

**Table 1:** Stern–Volmer constant (K_SV_) and dissociation constant (K_d_) obtained by fluorescence quenching analysis of PDIA1 and PDIA3 in the presence of punicalin. Data were calculated from fluorescence quenching analysis using both proteins (0.1 × 10^−6^ M) in reducing conditions (pH 7.4, 25 °C) and increasing concentration of punicalin (up to 10 × 10^−6^ M). K_SV_ values are reported as mean and SEM of at least three independent experiments. The values of K_SV_ and K_d_ obtained for punicalagin are from Paglia et al. [40].

	K_SV_ (M^−1^ × 10^3^)	K_d_ (M × 10^−6^)
Punicalin		
PDIA1	64.0 ± 0.4	16.0
PDIA3	73.7 ± 0.7	14.0
Punicalagin ^1^		
PDIA1	97.9 ± 2.1	11.9
PDIA3	157.1 ± 1.9	10.0

^1^ From Paglia et al. [40].

**Table 2:** IC_50_ values obtained by the glutathione reduction assay and calculated by fitting the dose–response data reported in Figure 3. The values of IC_50_ obtained for punicalagin were from Paglia et al. [40].

	IC_50_ (M × 10^−6^)	95% Confidence (M × 10^−6^)
Punicalin		
PDIA1	58.3	48.5 to 73.6
PDIA3	10.7	9.5 to 12.1
Punicalagin ^1^		
PDIA1	6.1	5.5 to 6.7
PDIA3	1.5	1.3 to 1.6

^1^ From Paglia et al. [40].

**Table 3:** IC_50_ values obtained by the insulin turbidity assay and calculated by fitting the dose–response data reported in Figure 4.

	IC_50_ (M × 10^−6^)	95% Confidence (M × 10^−6^)
Punicalin		
PDIA1	>250	120 to 3560
PDIA3	28.7	25.0 to 33.3
Punicalagin		
PDIA1	13.7	11.9 to 15.8
PDIA3	8.8	7.7 to 10.1

## Data Availability

Data are contained within the article.
